# Testosterone reduces hippocampal synaptic damage in an androgen receptor-independent manner

**DOI:** 10.1530/JOE-23-0114

**Published:** 2023-12-13

**Authors:** Yizhou Zhang, Meiqin Chen, Huan Chen, Shixiong Mi, Chang Wang, Hongchun Zuo, Leigang Song, Juan Du, Huixian Cui, Sha Li

**Affiliations:** 1 Department of Human Anatomy, Hebei Medical University, Shijiazhuang, Hebei, China; 2 Neuroscience Research Center, Hebei Medical University, Shijiazhuang, Hebei, China; 3 Hebei Key Laboratory of Neurodegenerative Disease Mechanism, Hebei Medical University, Shijiazhuang, Hebei, China

**Keywords:** testosterone, androgen receptor-independent manner, synaptic damage, Erk1/2–CREB signaling pathway

## Abstract

Aging-related reduction in androgen levels may be a possible risk factor for neurodegenerative diseases and contribute to cognitive impairment. Androgens may affect synaptic function and cognition in an androgen receptor (AR)-independent manner; however, the mechanisms connecting theses effects are unknown. Therefore, we used testicular feminization mutation (*Tfm*) male mice, a model with AR mutation, to test the effects of testosterone on synaptic function and cognition. Our results showed that testosterone ameliorated spatial memory deficit and neuronal damage, and increased dendritic spines density and postsynaptic density protein 95 (PSD95) and glutamate receptor 1 (GluA1) expression in the hippocampus of Tfm male mice. And these effects of testosterone were not inhibited by anastrozole, which suppressed conversion of testosterone to estradiol. Mechanistically, testosterone activated the extracellular signal-related kinase 1/2 (Erk1/2) and cyclic adenosine monophosphate response element-binding protein (CREB) in the hippocampus of *Tfm* male mice. Meanwhile, Erk1/2 inhibitor SCH772984 blocked the upregulation of phospho-CREB, PSD95, and GluA1 induced by testosterone in HT22 cells pretreated with flutamide, an androgen antagonist. Collectively, our data indicate that testosterone may ameliorate hippocampal synaptic damage and spatial memory deficit by activating the Erk1/2–CREB signaling pathway in an AR-independent manner.

## Introduction

Androgen is the primary male sex hormone that is involved in male sexual development and the maintenance of normal reproductive functions (Matsumoto *et al.* 2013, Rey 2021). In addition, androgen plays crucial roles in multiple functions of the brain like cognitive function (Fang *et al.* 2017, Cai & Li 2020, Jaeger *et al.* 2020, Tan *et al.* 2021*a*). Age-related reduction in androgen levels may be associated with cognitive impairment in neurodegenerative disorders (Bianchi *et al.* 2020, Li *et al.* 2020). However, there is a pressing need to better understand the mechanisms elucidating the effects of androgen on cognition.

The androgen receptor (AR), which acts as a transcriptional regulator, mediates the classic actions of androgens. Following activation by androgens, AR binds to specific DNA response elements in target gene promoters leading to transcription, which usually results in alterations in mRNA and protein synthesis (Takayama 2017). However, many cellular responses to androgens do not require the genomic activity of AR. For instance, activation of β-catenin and loss of Pten have been demonstrated to act together to drive AR-independent castration-resistant prostate cancer (Patel *et al.* 2020), and testosterone is reported to downregulate the proliferation of human umbilical vein endothelial cells by inducing arrest at the G1 phase of the cell cycle in an AR-independent manner (Gaba *et al.* 2018). These androgen induced effects may be caused by the direct activation of intracellular kinase cascades independent of AR. In addition, testosterone, the major androgen in men, can be converted into estradiol by aromatase, which then exerts biological effects through the estrogen receptor (ER). Several case reports suggest that men with mutations in the ER or aromatase genes may have severe osteoporosis, indicating that, to some extent, the activities of testosterone are mediated by its aromatization to estradiol (Mohamad *et al.* 2016). It is worth noting that, to date, the roles and mechanisms of androgen AR-independent pathway in cognitive function have not been fully clarified.

The contribution of androgen to spatial memory is related to hippocampal synaptic function (Neves *et al.* 2008, Platholi & Hemmings 2021). Synaptic damage in the hippocampus, a crucial component of the medial temporal lobe memory circuit, is thought to be one of the early changes that occur in AD against a pathological background of extracellular amyloid-beta deposition and intracellular neurofibrillary tangle formation (Mufson *et al.* 2015, Styr & Slutsky 2018). Androgen deficiency impairs cognitive function by inducing the hippocampal synaptic damage (Shelton & Rajfer 2012, Tan *et al.* 2021*a*). As per a study, after gonadectomy, fewer mushroom-type and more stubby- and thin-type spines were observed in pyramidal neurons in the hippocampal CA1 subregion, and this effect was prevented by testosterone replacement (Li *et al.* 2012). Testosterone administration also promoted the synaptic plasticity-related proteins expression, such as synapsin 1 (SYN1, a presynaptic marker) and PSD95 (a postsynaptic marker) (Fang *et al.* 2017, Yan *et al.* 2019).

To study the AR-independent role of androgens on spatial memory, we used testicular feminization mutation (*Tfm*) male mice, an animal model harboring an AR gene mutation with a dominant spontaneous mutation on the X chromosome (Lyon & Hawkes 1970). *Tfm* male mice (hemizygous, X*^Tfm^* Y) are outwardly female in appearance and due to abnormal development of their reproductive system, the testes are very small. Leydig cells of their testes fail to develop normally, resulting in a significant reduction in androgen levels (Murphy *et al.* 1994). Meanwhile, Rizk *et al.* (2005) reported that *Tfm* male mice have spatial memory deficits. In this study, we found that testosterone administration ameliorated the synaptic damage and spatial memory deficit, which the aromatase inhibitor anastrozole could not inhibit, indicating that testosterone could directly improve these functions in an AR-independent manner. Moreover, testosterone activated the extracellular signal-related kinase 1/2 (Erk1/2)-cyclic adenosine monophosphate response element-binding protein (CREB) signaling pathway in the hippocampus of *Tfm* male mice. Meanwhile, inhibition of Erk1/2 phosphorylation blocked the increased expression of synaptic plasticity-related proteins by testosterone in HT22 cells pretreated with an androgen antagonist flutamide. HT22 is a hippocampal neuronal cell line that has been used as a good model for memory-related studies because it is capable of mimicking long-term potentiation without establishing synaptic connections (Morimoto & Koshland 1990). These findings supported the concept that androgen may affect spatial memory and hippocampal synaptic function via the Erk1/2–CREB signaling pathway in an AR-independent manner.

## Materials and methods

### Animals and treatments

Vital River Laboratory Animal Technology Co., Ltd. (Beijing, China) provided C57BL/6J male mice, and *Tfm* mice (strain #: 000569) were obtained from Jackson Laboratory. Mice were housed at the Center of Laboratory Animal Research and Service of Hebei Medical University under conditions of constant temperature (22 ± 2°C), humidity (55 ± 5%), and lighting (12-h light–12-h darkness cycle). All animal experiments in this study were complied with the guidelines of the Animal Welfare Act of the National Institutes of Health Guide for the Care and Use of Laboratory Animals (NIH Publication No. 85-23, revised 1996), and were approved by the Ethics Committee of Hebei Medical University (IACUC-Hebmu-Glp-2,016,017).

We used six-month-old mice in the experiment according to Angela Rizk’s research (Rizk *et al.* 2005). Male mice were used throughout this study. Six-month-old mice were randomly divided into four groups: WT group, littermate wild-type male mice; *Tfm* group, *Tfm* male mice; *Tfm*+T group, *Tfm* male mice treated with testosterone (0.6 mg/kg/day, i.p., Cat. No. T0027, KITA-KU, Tokyo, Japan); *Tfm*+A+T group, *Tfm* male mice treated with testosterone combined with anastrozole (10 mg/kg/day, i.p., Cat. No. B1382, APExBIO, Houston, TX, USA). Testosterone and anastrozole were dissolved in corn oil. Mice in WT and *Tfm* groups were treated with an equivalent volume of corn oil. All animals received the indicated treatments daily for 30 days.

### Cells and treatments

Pricella (Wuhan, China) provided immortalized mouse hippocampal HT22 cells (Cat. No. CL-0697). Cells were maintained in Dulbecco’s modified Eagle’s medium with 10% fetal bovine serum and a 0.2% penicillin/streptomycin solution in humidified atmosphere saturated with 5% CO_2_ at 37°C. When 70–80% confluence was attained, the cells were dissociated from culture dishes using 0.25% trypsin at 37°C. Cells were seeded at 50,000 cells/cm^2^ and cultured for another day in 6-well plates for Western blot or mounted on coverslips in 12-well plates for immunofluorescence staining. To reduce the effect of AR, all cells were pretreated with its antagonist, flutamide (F, 10 μM, Cat. No. F0663, Tokyo Chemical Industry Co., Ltd, Japan) for 1 h. Cells were then randomly divided into four groups: F+DMSO (dimethyl sulfoxide) group; F+T group, cells treated with testosterone (10 nM); F+SCH772984 group, cells treated with SCH772984 (100 nM, Cat. No. S7101, Selleck Chemicals, TX, USA); F+SCH772984+T group, cells treated with SCH772984 for 2 h and then with testosterone. Testosterone, flutamide, and SCH772984 were dissolved in DMSO. Cells in F+T and F+SCH772984+T groups were treated with testosterone for 48 h. Other groups were treated with an equivalent volume of DMSO.

### Sex determination PCR and gene sequencing

Genomic DNA was isolated from the animals’ tails using the One Step Mouse Genotyping Kit (Cat. No. PD101–01, Vazyme Biotech Co., Ltd, Nanjing, China). Detection of the Y chromosome was performed using PCR with 2× Taq PCR MasterMix (Cat. No. KT201, TIANGEN, Beijing, China). The samples were incubated at 94°C for 3 min, followed by 35 cycles of denaturation at 94°C for 30 s, annealing at 55°C for 30 s, and cDNA extension at 74°C for 30 s. After the amplification cycles, a final extension step at 72 °C was performed for 4 min. Mice were genotyped by sex determination PCR analysis for detecting the Y chromosome which was amplified using the following primers: forward, 5′-ACTTGTCTTGCCATTCTGTT-3′, and reverse 5′-CTCCCATCCTTCTAATCTGT-3′. The AR mutation site was detected using first-generation sequencing techniques (Sangon Biotech, Shanghai, China). AR was amplified using the following primers: forward, 5′-CTTTCAAGGGAGGTTACGC-3′, and reverse, 5′-CCAGGAAGAACAGGTGGTG-3′.

### Morris water maze test

The Morris water maze test was performed in a pool (diameter 120 cm, height 60 cm) filled with water maintained at room temperature (23 ± 1°C) and made opaque with nontoxic white paint. The pool was located in a separate room that contained visible cues and virtually divided into four equal quadrants. To escape the test situation, the mice (*n* = 6 for each group) had to reach a hidden platform (diameter 10 cm) that was located in the middle of one fixed quadrant and submerged 1 cm under the water surface. After a day of habituation, mice were tested in four trials per day for five consecutive days from each starting point in a random order. Each trial had a 60 s time limit. Mice were gently guided to the platform by hand if they failed to reach the target within 60 s. The escape latency to the hidden platform was recorded. Twenty-four hours after the last training trial (day 6), the platform was removed, and mice were given one probe trial in which they were allowed to search the platform for 60 s (probe test). The number of times the animal crossed the platform area, the latency to the first entrance to the target, and the average swimming speed were recorded.

### Hematoxylin–eosin staining

To observe the morphology of the hippocampus, hematoxylin–eosin (H&E) staining was performed. Two days after the behavior tests, mice (*n* = 4 for each group) were sacrificed and the brain tissues were collected. Paraffin sections (5 μm) were prepared after perfusing tissues with 4% paraformaldehyde. Each section was dewaxed twice in xylene (10 min each time) and rehydrated by passage through a graded alcohol series (5 min each time). Sections were stained with hematoxylin solution for 5 min, rinsed in distilled water for 1 min and differentiated in 1% hydrochloric acid ethanol for 3 s. The sections were then stained with eosin solution for 2 min followed by dehydration by passage through a graded alcohol series. Images of the hippocampal CA1, CA3, and DG subregions were then captured.

### Nissl staining

Following deparaffinization and dehydration, the paraffin sections (5 μm, *n* = 4 for each group) were stained with 1% cresyl violet at 60°C for 30 min, followed by washing with distilled water. The sections were then differentiated in 95% ethanol, dehydrated with 100% ethanol, cleared in xylene, and mounted on coverslips using a resinous mounting medium. Then photos of the slices were taken under a microscope at 40× magnification.

### Golgi–Cox staining

After removal of the brain, the hemispheres (*n* = 5 for each group) were immersed in Reagent A (10 mL) + Reagent B (10 mL) solution from the Golgi Stain Kit (GenMED Scientifics Inc., Arlington, MA, USA). The brain tissue samples were incubated for 14 days at room temperature in darkness, following which they were then immersed in 30% sucrose solution for 48 h in the dark and 100 μm sections obtained using a vibratome. The sections were mounted onto slides and air-dried for 24 h in darkness. The slides on which the sections had been mounted were rinsed three times with Reagent G (3 min for time), and then transferred to Reagent H for 30 min. After rinsing, the slides were immersed in a mixture of 10 mL Reagent I + 10 mL Reagent J for 20 min. The slides were then dehydrated by passage through a graded ethanol series and mounted on coverslips using Permount. All slides were blinded, and neurons in the sections were imaged. For each mouse, five randomly chosen, representative neurons from five sections were analyzed and the average values for each mouse were presented in the results. The dendritic spine density was quantified using FiJi/Image J (National Institutes of Health).

### Western blot analysis

Mice hippocampi or HT22 cells (*n* = 5 for each group) were dissected and lysed in Radio Immunoprecipitation Assay buffer containing 300 μL PMSF, 1× protease inhibitor cocktail and 1× phosphatase inhibitor cocktail. The lysates were boiled at 95°C in 5× loading buffer for 5 min and stored at −80°C. A total of 20 μg protein per sample was resolved by SDS-PAGE (5%) at 80 V and then SDS-PAGE (10%) at 120 V. The gel proteins were transferred onto PVDF membranes (Millipore), blocked using 5% milk solution for 1 h at room temperature, and then incubated overnight with rabbit anti-PSD95 polyclonal antibody (1:1000, Cat. No. ab18258, Abcam), rabbit anti-GluA1 monoclonal antibody (1:1000, Cat. No. ab183797, Abcam), mouse anti-phospho-Erk1/2 monoclonal antibody (1:1000, Cat. No. 9106, Cell Signaling Technology), mouse anti-total-Erk1/2 monoclonal antibody (1:1000, Cat. No. 4696, Cell Signaling Technology), mouse anti-phospho-CREB monoclonal antibody (1:1000, Cat. No. 9196, Cell Signaling Technology), mouse anti-total-CREB monoclonal antibody (1:1000, Cat. No. 9104, Cell Signaling Technology), or rabbit anti-β-actin polyclonal antibody (1:1000, Cat. No. 20536-1-AP, Proteintech) at 4°C. After washing with TBST three times (5 min each time), the membranes were blotted with secondary antibody (anti-rabbit IgG Dylight™ 800 Antibody, Cat. No. 611-145-002 and anti-mouse IgG Dylight™ 680 Antibody, Cat. No. 610-144-002, 1:10,000, ROCKLAND) for 2 h at room temperature. The membranes were washed again and the reactions visualized and analyzed using an Odyssey IR fluorescence scanning imaging system (LI-COR, USA).

### Immunohistochemistry

Following deparaffinization and dehydration, the paraffin sections (5 μm, *n* = 4 for each group) were subjected to antigen retrieval in a pressure cooker for 5 min. The sections were then immersed in 3% hydrogen peroxide in methanol for 30 min to stop the endogenous peroxidase activity. Subsequently, sections were incubated with 5% normal goat serum to block nonspecific binding, followed by an overnight incubation with rabbit anti-PSD95 polyclonal antibody (1:200, Cat. No. ab18258, Abcam), rabbit anti-GluA1 monoclonal antibody (1:100, Cat. No. ab183797, Abcam), mouse anti-phospho-Erk1/2 monoclonal antibody (1:200, Cat. No. 4370, Cell Signaling Technology) and mouse anti-phospho-CREB monoclonal antibody (1:200, Cat. No. 9198, Cell Signaling Technology) at 4°C. After being washed, the sections were incubated with biotinylated goat rabbit anti-IgG for 1 h. Reactants were visualized with DAB Detection Kit (ZLI-9019, ZSBIO) for 3 to 5 min. Hematoxylin somatic cell staining reagent was used to counterstain nuclei. Sections were sealed with neutral resin. Images of the hippocampal CA1, CA3, and DG regions were captured at 20× magnification in a single plane and analyzed using Image-Pro Plus 6.0 (Media Cybernetics, Rockville, MD, USA).

### Immunofluorescence staining

A treatment with ice-cold 4% PFA for 10 min at room temperature was used to fix cells (*n* = 3 for each group). After washing with 0.01 M PBS, cells were blocked with PBS-T (0.5% Triton X-100) containing 5% donkey serum for 1 h at room temperature, followed by an overnight incubation with rabbit anti-PSD95 polyclonal antibody (1:200, Cat. No. ab18258, Abcam), mouse anti-GluA1 monoclonal antibody (1:200, Cat. No. ab174785, Abcam), rabbit anti-phospho-Erk1/2 monoclonal antibody (1:200, Cat. No. 4370, Cell Signaling Technology), rabbit anti-phospho-CREB monoclonal antibody (1:200, Cat. No. 9198, Cell Signaling Technology) at 4°C. Cells were then washed and incubated with secondary antibodies (Donkey anti-Rabbit IgG (H+L) Highly CrossAdsorbed Secondary antibody Alexa Fluor® 594, 1:1,000, cat#: A-21207, and Donkey anti-Mouse IgG (H+L) Highly Cross-Adsorbed Secondary Antibody, Alexa Fluor™ 594, 1:1,000, cat#: A-21203, Thermo Scientific™) for 2 h at room temperature. Thereafter, diamidino-phenyl-indole (DAPI, 1:200, Cat. No. C0060, Solarbio) was added for 10 min. Images were acquired with an inverse Olympus FV1200 microscope equipped with a corresponding fluorescence system and the intensity measured using Image Pro Plus 6.0.

### Statistical analysis

All data were analyzed using SPSS 23.0 statistical software (SPSS Inc., Chicago, IL, USA). The latency-to-escape data from the Morris water maze test were analyzed by repeated measures ANOVA. The data on the numbers of platform crossing were evaluated using the Kruskal–Wallis *H* test. For other data, one-way ANOVA (Dunnett’s *post* *hoc* test for multiple comparisons) was used. The results are expressed as the mean ± SEM. Statistical significance was defined as *P < 0.05, **P < 0.01, ***P < 0.001.

## Results

### Testosterone ameliorated spatial memory impairment in *Tfm* male mice

**Figure 1:**
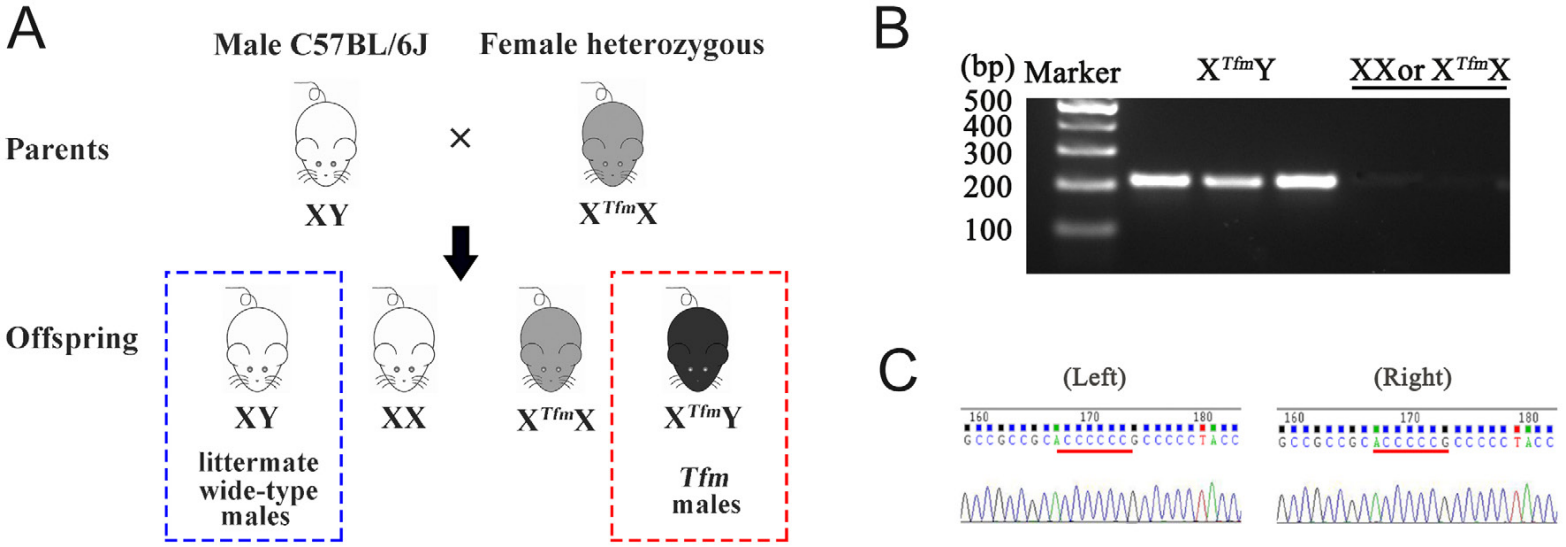
Reproduction and identification of *Tfm* male mice. (A) We crossed the female *Tfm* heterozygous mice (X*^Tfm^*X, carrying one Tfm allele and one wild-type of the androgen receptor) with wild-type male C57BL/6J mice to obtain Tfm male mice (X*^Tfm^*Y, completely androgen insensitive). (B) Gender-determination PCR for the Y chromosome to distinguish *Tfm* male mice from the female *Tfm* heterozygous mice (X*^Tfm^*X) and the female wild-type mice (XX). (C) Sequence of AR DNA from littermate wild-type male mice (left) and *Tfm* male mice (right). The red horizontal bar indicates the site of the AR mutation. A full color version of this figure is available at https://doi.org/10.1530/JOE-23-0114.

To test whether testosterone impacts spatial memory in an AR-independent manner, we used Tfm male mice (X*^Tfm^*Y) as an animal model (Fig. 1A, red dashed box) and littermate wild-type male mice as control (Fig. 1A, blue dashed box). Though *Tfm* male mice are outwardly female in appearance, they still have Y chromosome. Thus, we used gender-determination PCR for the Y chromosome to distinguish *Tfm* male from the female *Tfm* heterozygous (X*^Tfm^*X) and the female wild-type mice (Fig. 1B). PCR-amplified products that were sequenced directly showed that the cDNA sequence of wild-type AR is CACCCCCCG, whereas the sequence of the same region in the *Tfm* male mice is CACCCCCG (Fig. 1C). This mutation in the DNA sequence results in loss of the normal function of AR.

**Figure 2:**
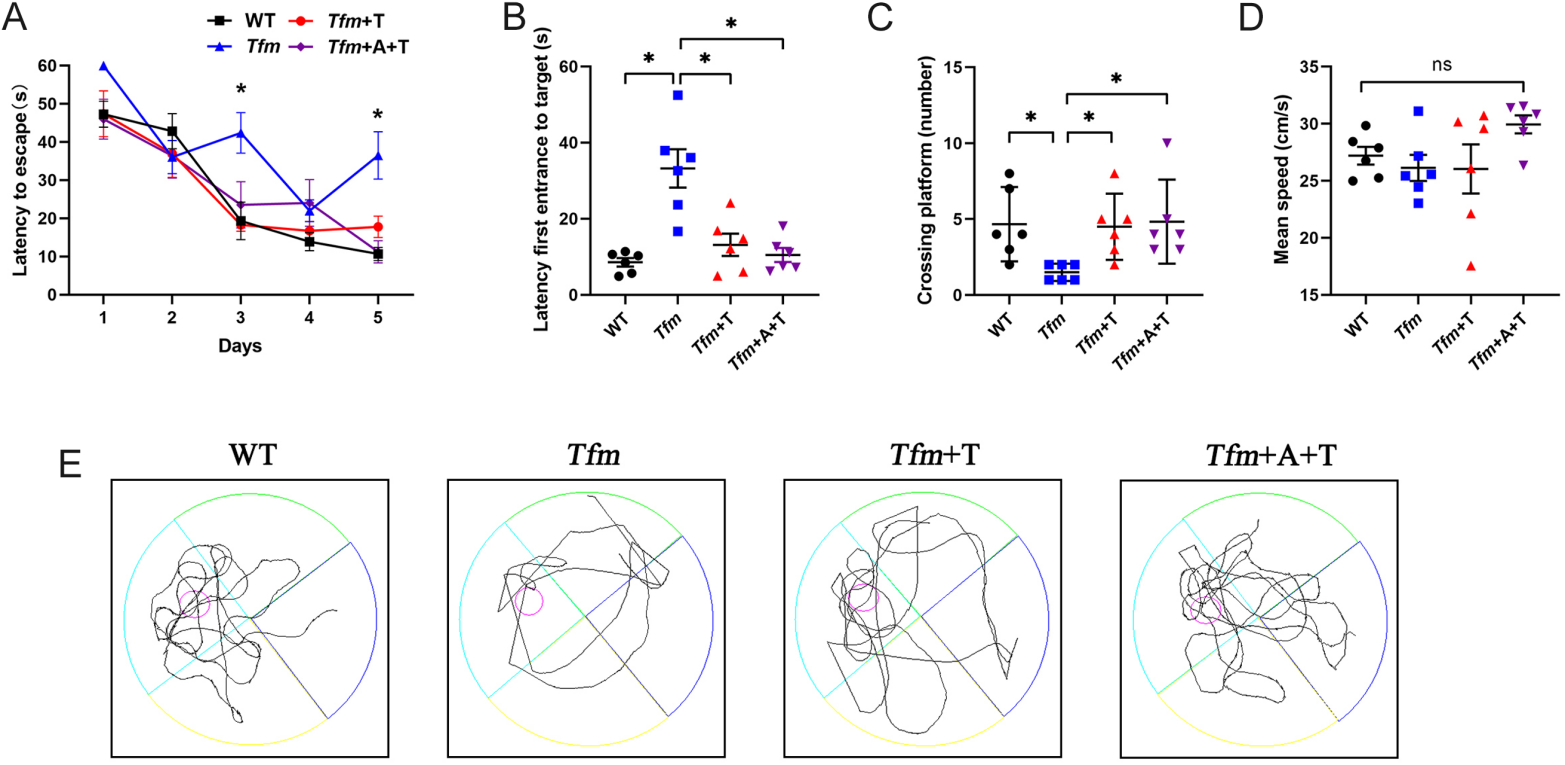
Effects of testosterone on spatial memory in *Tfm* male mice. (A) Mean escape latency to the hidden platform in the Morris water maze test. (B) Latency to find the hidden platform area for the first time in the Morris water maze probe test. (C) Numbers of platform location crossings in the Morris water maze probe test. (D) Mean speed of each group in the Morris water maze probe test. (E) Swimming paths of each group in the Morris water maze probe test. *n* = 6. *P < 0.05 vs *Tfm* group, ns: not significant. A full color version of this figure is available at https://doi.org/10.1530/JOE-23-0114.

The Morris water maze task was used to evaluate spatial memory and the results showed that Tfm group spent more time navigating to the platform on days 3 and 5 in hidden platform training than WT group, *Tfm*+T group, and *Tfm*+A+T group (Fig. 2A). In the probe test, the mice in the *Tfm* group displayed a significant increase in the latency to find the hidden platform area for the first time (Fig. 2B) and a significant decrease in the numbers that crossed the platform location (Fig. 2C) compared with other groups. There were no significant differences in average swimming speed among these groups (Fig. 2D). The swimming trials of the mice in each group in the probe test are shown in Fig. 2E. Collectively, the data suggested that testosterone ameliorated the spatial memory deficits of *Tfm* male mice in an AR-independent manner.

### Testosterone reduced hippocampal neuronal damage and loss in *Tfm* male mice

**Figure 3:**
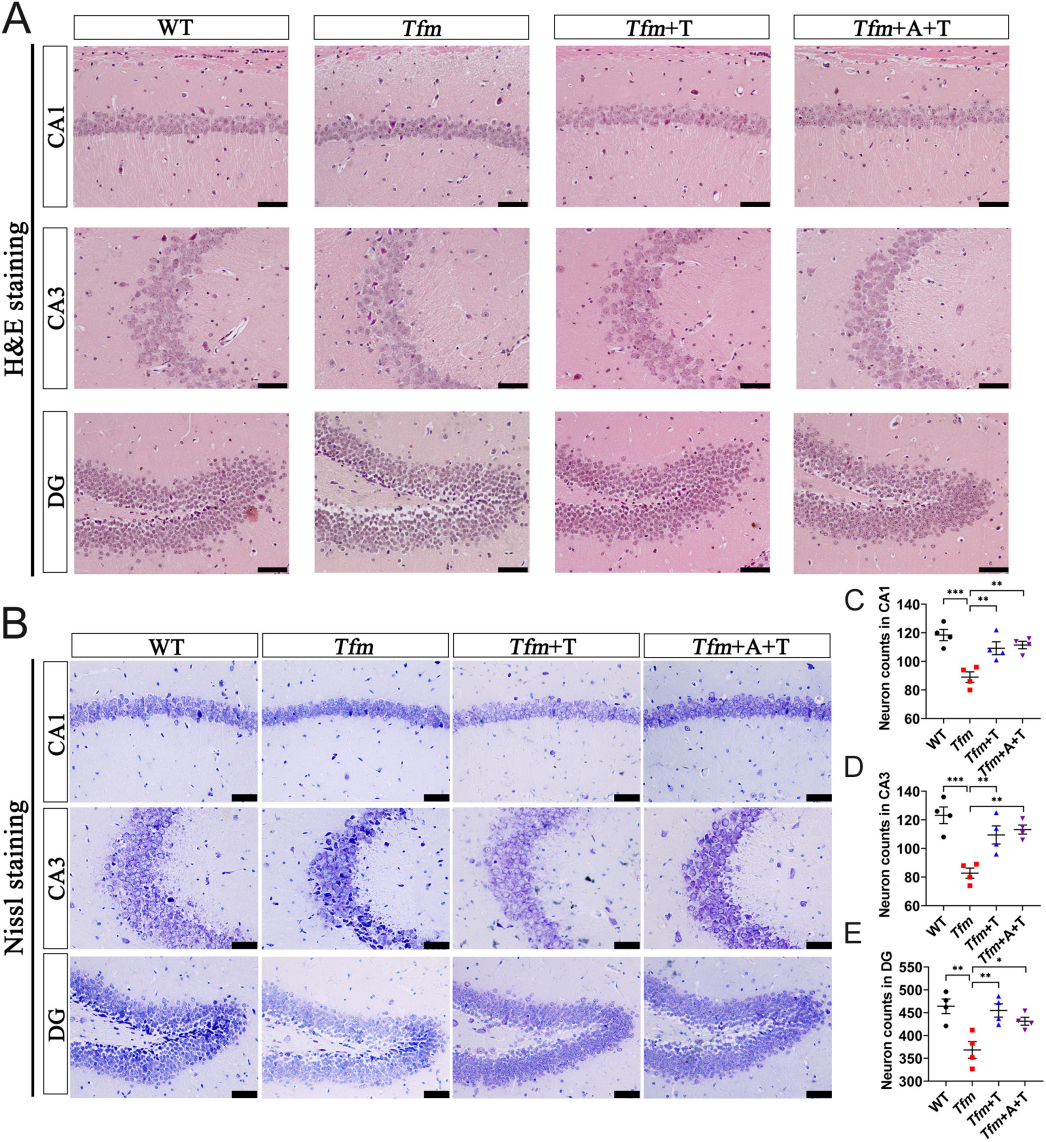
Effects of testosterone on hippocampal neuronal damage and loss in the hippocampus of *Tfm* male mice. (A) Hematoxylin and eosin staining in the hippocampal CA1, CA3, and DG subregions. (B–E) Nissl staining in the hippocampal CA1, CA3, and DG subregions. *n* = 4, scale bar = 50 μm. *P < 0.05, **P < 0.01, ***P < 0.001 vs *Tfm* group. A full color version of this figure is available at https://doi.org/10.1530/JOE-23-0114.

The effect of testosterone on neuronal damage and loss was determined using H&E and Nissl staining. The results showed that the tissue structure and neuronal morphology of the hippocampal CA1, CA3, and DG subregions in the WT group were arrayed regularly and compactly. However, the tissue structure and neuronal morphology of the same regions in the *Tfm* group were incomplete and disordered, and the cell nuclei were either segmented or absent. Testosterone administration (*Tfm*+T group) reduced the neuronal damage, and anastrozole (*Tfm*+A+T group) did not alter this effect (Fig. 3A). Moreover, compared with the WT group, Nissl staining revealed a significant neuronal loss in hippocampal CA1, CA3, and DG subregions in *Tfm* male mice, with some neurons stained darker. Following administration of testosterone (*Tfm*+T group), the decrease of Nissl-positive neurons was ameliorated, and anastrozole (*Tfm*+A+T group) did not alter this effect of testosterone (Fig. 3B, C, and D). These findings indicated that testosterone had a protective effect on hippocampal neurons through an AR-independent pathway.

### Testosterone increased hippocampal dendritic spine density and PSD95 and GluA1 expression in *Tfm* male mice

**Figure 4:**
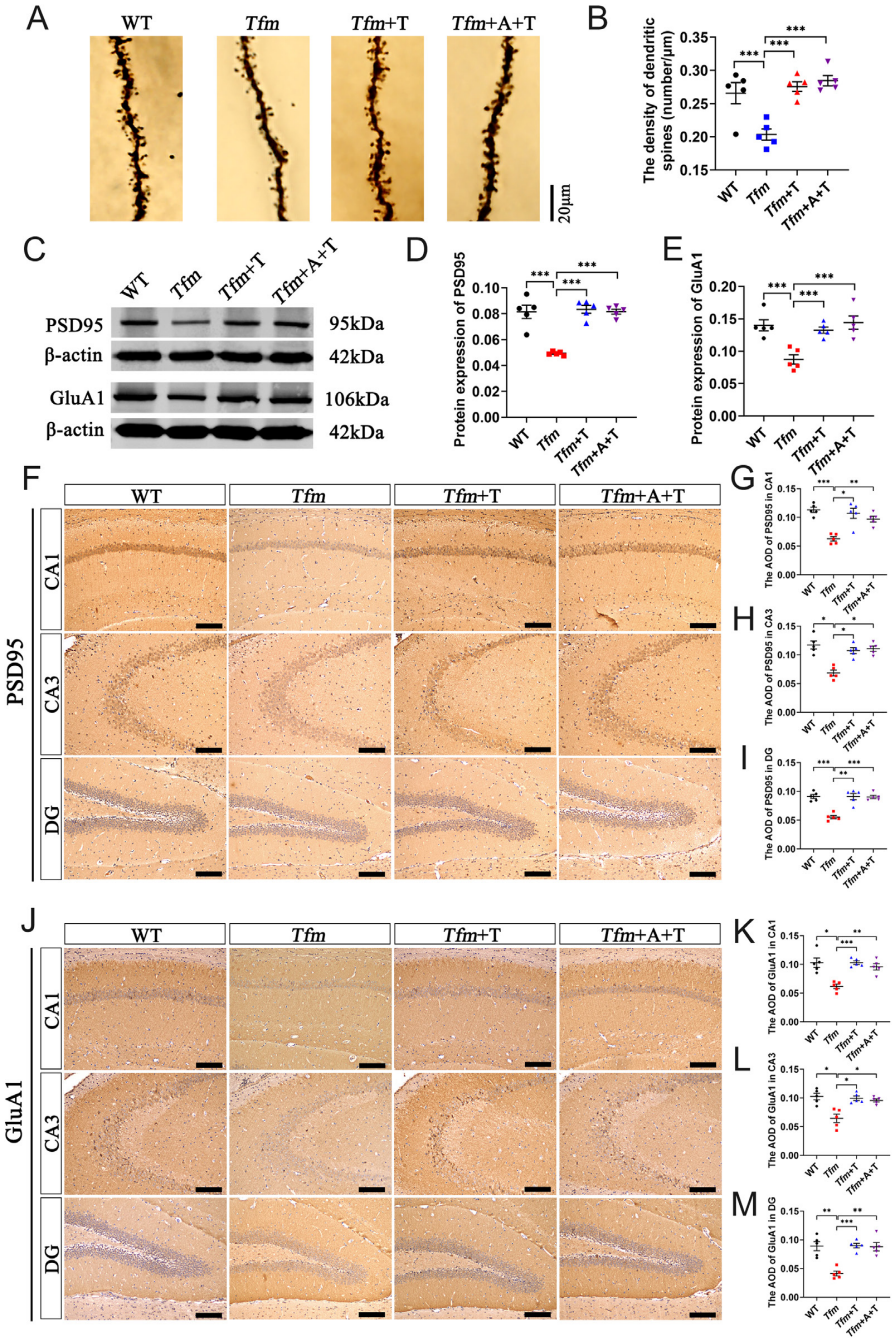
Effects of testosterone on the hippocampal synaptic function of *Tfm* male mice. (A, B) Golgi–Cox staining of hippocampal neurons (*n* = 5, scale bar = 20 μm). (C–E) Western blot analysis of PSD95 and GluA1 protein levels in the hippocampus (*n* = 5). (F–I) Immunohistochemistry of PSD95 protein level in the hippocampal CA1, CA3, and DG subregions (*n* = 5, scale bar = 100 μm). (J–M) Immunohistochemistry of GluA1 protein level in the hippocampal CA1, CA3, and DG subregions (*n* = 5, scale bar = 100 μm). *P < 0.05, **P < 0.01, ***P < 0.001 vs *Tfm* group. A full color version of this figure is available at https://doi.org/10.1530/JOE-23-0114.

We profiled synaptic plasticity-related changes at the structural level in the hippocampus using Golgi–Cox analysis. It was observed that the dendritic spine density of hippocampal pyramidal neurons in *Tfm* group was reduced compared with WT group. Testosterone administration (*Tfm*+T group) eliminated the decrease of the dendritic spine density, while anastrozole (*Tfm*+A+T group) did not alter this effect (Fig. 4A and B). We then assessed synaptic plasticity-related protein PSD95 and GluA1 expression through Western blot and immunohistochemistry. PSD95, a scaffolding protein, plays a crucial role as it organizes key postsynaptic density components essential for synaptic plasticity, signaling, development, and survival (Levy *et al.* 2022) and GluA1 is a subunit of α-amino-3-hydroxy-5-methyl-4-isoxazole propionic acid (AMPA) receptor that mediates majority of fast excitatory synaptic transmission in the brain (Diering & Huganir 2018, Qu *et al.* 2021). Western blot analysis showed that there were significantly lower levels of PSD95 and GluA1 proteins in the hippocampus of *Tfm* group compared with WT group. Testosterone administration (*Tfm*+T group) abrogated the downregulation of these two proteins, and anastrozole (*Tfm*+A+T group) had no such downregulation effects (Fig. 4C, D, and E). Consistently, immunohistochemistry of hippocampal CA1, CA3, and DG subregions showed that the expression of PSD95 and GluA1 in *Tfm* group were lesser than WT group. Testosterone administration (*Tfm*+T group) increased the levels of these two proteins in same regions and anastrozole (*Tfm*+A+T group) did not affect these effects (Fig. 4F, G, H, I, J, K, L, and M). Collectively, these results indicated that testosterone enhanced hippocampal synaptic plasticity in Tfm male mice via an AR-independent pathway.

### Testosterone activated the Erk1/2–CREB signaling pathway in *Tfm* male mice

**Figure 5:**
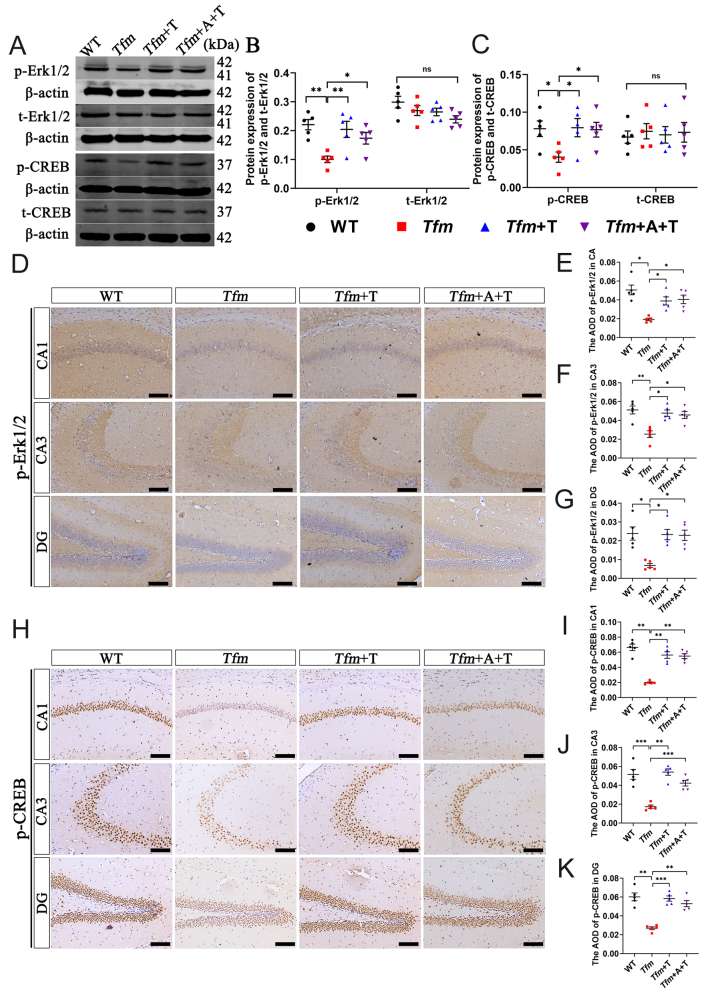
Effects of testosterone on the Erk1/2–CREB signaling pathway in the hippocampus of Tfm male mice. (A–C) Western blot analysis of p-Erk1/2, t-Erk1/2, p-CREB, and t-CREB protein levels in the hippocampus (*n* = 5). (D–G) Immunohistochemistry of p-Erk1/2 protein level in the hippocampal CA1, CA3, and DG subregions (*n* = 5, scale bar = 100 μm). (H–K) Immunohistochemistry of p-CREB protein level in the hippocampal CA1, CA3, and DG subregions (*n* = 5, scale bar = 100 μm). *P < 0.05, **P < 0.01, ***P < 0.001 vs *Tfm* group. A full color version of this figure is available at https://doi.org/10.1530/JOE-23-0114.

Several signal transduction pathways activated by testosterone via AR-independent mechanisms, including c-Src, have been reported in prostate cancer cells. c-Src is a nonreceptor tyrosine kinase that regulates a complex signaling network that drives prostate tumor progression (Kim *et al.* 2019). Following activation by androgens, Src mediates cell function through the MAPK–Erk1/2–CREB pathway (Cheng *et al.* 2007, Zarif *et al.* 2015). Thus, we used Western blot and immunohistochemistry to evaluate the role of the Erk1/2–CREB signaling pathway in the AR-independent androgen effect. As expected, results of the Western blot showed that p-ERK1/2 and p-CREB expression in the *Tfm* group decreased compared with WT group. Testosterone administration (*Tfm*+T group) increased the level of these proteins and anastrozole (*Tfm*+A+T group) did not reduce these effects. However, there were no differences in t-Erk1/2 and t-CREB expression among groups (Fig. 5A, B, and C). Immunohistochemistry results also showed that expression of p-Erk1/2 and p-CREB in CA1, CA3, and DG subregions of *Tfm* mice were less than WT group, but testosterone treatment (*Tfm*+T group) increased the levels of p-Erk1/2 (Fig. 5D, E, F, G, and H) and p-CREB (Fig. 5H, I, J, and K). Overall, these results supported the view that testosterone could affect hippocampal synaptic function and spatial memory of *Tfm* male mice via the Erk1/2–CREB signaling pathway in an AR-independent manner.

### Erk1/2 activation is required in testosterone-induced CREB phosphorylation in HT22 cells

**Figure 6:**
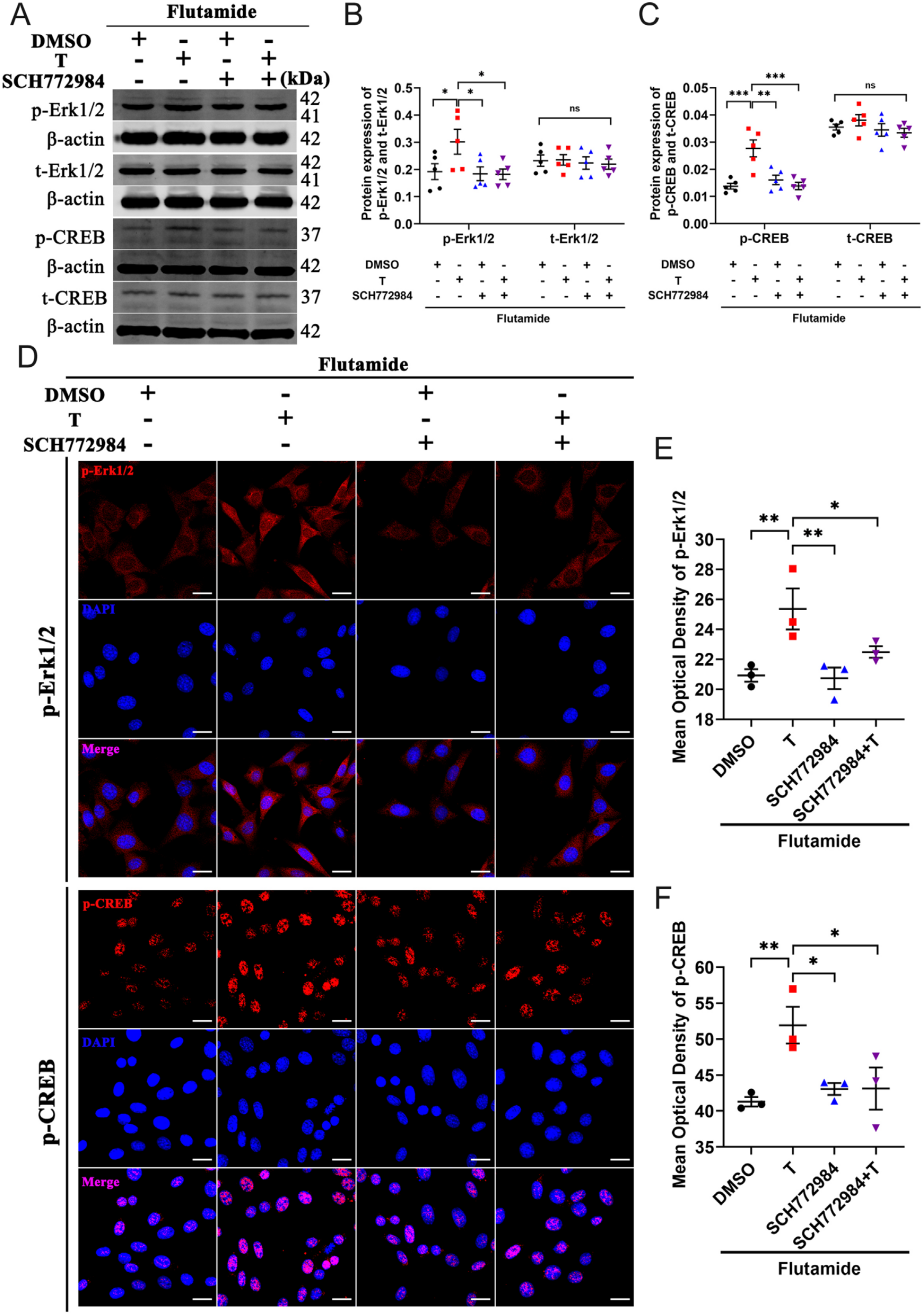
Erk1/2 activation is required in testosterone-induced CREB phosphorylation in HT22 cells pretreated with flutamide. (A–C) Western blot analysis of p-Erk1/2, t-Erk1/2, p-CREB, and t-CREB protein levels in HT22 cells pretreated with flutamide (*n* = 5). (D–F) Immunofluorescence staining of p-Erk1/2 and p-CREB proteins in the HT22 cells pretreated with flutamide (*n* = 3, scale bar=20 μm). *P < 0.05, **P < 0.01, ***P < 0.001 vs *Tfm* group. A full color version of this figure is available at https://doi.org/10.1530/JOE-23-0114.

To further clarify the role of the Erk1/2–CREB signaling pathway in the AR-independent manner, AR-independent response, we pretreated HT22 cells with flutamide, an AR antagonist, and then incubated them with Erk1/2 inhibitor SCH772984. The results of Western blot analysis showed that even when AR was blocked, testosterone could lead to phosphorylation/activation of Erk1/2. In addition, SCH772984 effectively inhibited testosterone induced Erk1/2 phosphorylation/activation compared with the T+F group (Fig. 6A and B). Similar results were obtained in the immunofluorescence assay (Fig. 6D and E). Western blot and immunofluorescence analysis showed that Erk1/2 inhibition blocked its downstream CREB phosphorylation/activation induced by testosterone (Fig. 6A, C, D, E, and F).

### Erk1/2 activation is required in testosterone-induced PSD95 and GluA1 expression in HT22 cells

**Figure 7:**
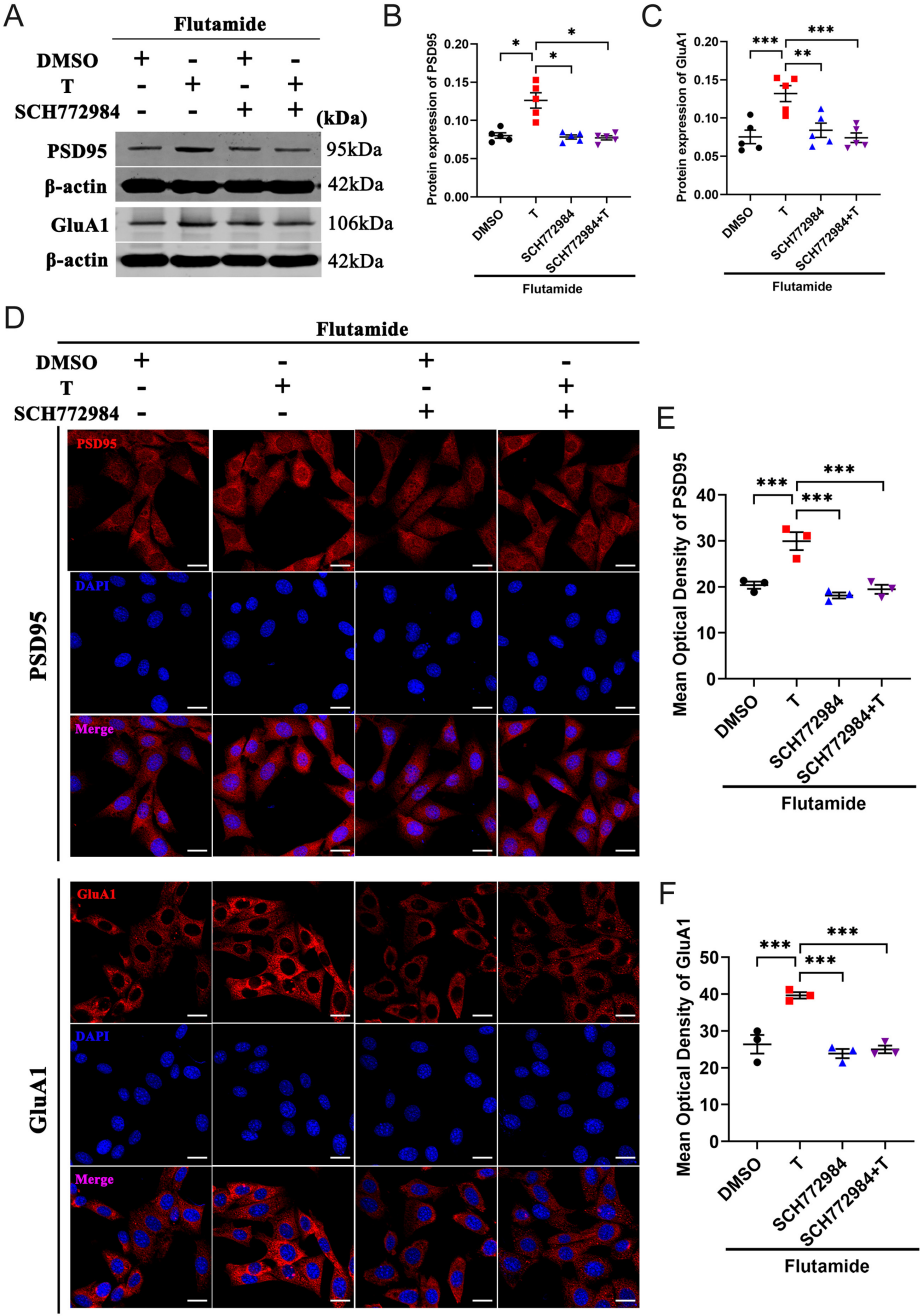
Erk1/2 activation is required in testosterone-induced PSD95 and GluA1 expression in HT22 cells pretreated with flutamide. (A–C) Western blot analysis of PSD95 and GluA1 protein levels in HT22 cells pretreated with flutamide (*n* = 5). (D–F) Immunofluorescence staining of PSD95 and GluA1 proteins in the HT22 cells pretreated with flutamide (*n* = 3, scale bar = 20 μm). *P < 0.05, **P < 0.01, ***P < 0.001 vs *Tfm* group. A full color version of this figure is available at https://doi.org/10.1530/JOE-23-0114.

Next, to elucidate the role of the Erk1/2–CREB signaling pathway on synaptic function, we pretreated HT22 cells with flutamide, and then incubated them with testosterone in presence and absence of Erk1/2 inhibitor SCH772984. Western blot and immunofluorescence assay were also used to detect the expression of PSD95 and GluA1 proteins in HT22 cells. The results of Western blot analysis showed that SCH772984 effectively inhibited testosterone induced the expression of PSD95 and GluA1 compared with testosterone combined with flutamide (T+F) group (Fig. 7A, B, and C). Similar results were obtained in immunofluorescence assay (Fig. 7D, E, and F). These results further demonstrate the role of the Erk1/2–CREB signaling pathway in testosterone-induced synaptic plasticity through an AR-independent manner.

## Discussion

The androgens classically are thought to play a biological role by activating the transcription factor activity of the ARs. However, numerous studies have reported that many cellular responses to androgens are not diminished by transcription inhibitors, indicating that these actions may be independent of the ligand dependent transactivation function of AR (Zamagni *et al.* 2019). Here, we found that *Tfm* male mice, an animal model with AR mutation, exhibited defects in spatial memory, which may be due to decreases in the androgen levels or the AR dysfunction. Rizk *et al.* (2005) also reported that *Tfm* male mice had spatial memory deficits and showed that *Tfm* female mice (heterozygous, X*^Tfm^*X) outperformed *Tfm* male mice in the water maze test. Significantly, in this study, we demonstrated that testosterone supplementation improved spatial memory impairment in *Tfm* male mice, indicating that testosterone may also affect the nervous system functions in an AR-independent manner. However, the AR gene mutation may cause developmental issues, and it is a global mutation but not limited to the hippocampus. Thus, the AR gene mutation may cause a no specific effect on cognition through other mechanisms. For instance, Brooks *et al.* (2020) reported that testosterone partially activated male behavior by aromatizing into estradiol in the brain. However, the results of this study showed that aromatase inhibition did not block the positive effects of testosterone on spatial memory of *Tfm* male mice. These findings suggest that testosterone might directly affect spatial memory in an AR-independent manner.

Sex steroid hormones including androgens, estrogens, and progesterone are involved in learning and memory (Colciago *et al.* 2015). Synaptic plasticity, an activity-dependent change in neuronal connection strength, is a biological process that is speculated to contribute to learning and memory (Citri & Malenka 2008, Magee & Grienberger 2020). Murakami *et al.* (2018) reported that 10 nM testosterone increased the dendritic spine density in the hippocampal CA1 subregion within 2 h via AR in acute hippocampal slices from adult male rats. Meanwhile, Ziehn *et al.* (2012) reported that testosterone administration preserved excitatory synaptic transmission in the hippocampus. Our results showed that testosterone administration also increased dendritic spine density and PSD95 and GluA1 protein levels in the hippocampus of *Tfm* male mice with AR mutation, suggesting that it may also affect the hippocampal synaptic function via an AR-independent pathway. Moreover, we also found that testosterone supplementation reduced neuronal damage in *Tfm* male mice. Consistent with our results, Muthu and Seppan (2020) demonstrated that testosterone levels are a potential cause of cognitive impairment induced by irreversible neuronal damage. Thus, testosterone may have a neuroprotective effect that is exerted in an AR-independent manner.

A description of kinase-dependent signaling mechanisms is provided that could partially explain the AR-independent effects of testosterone on the spatial memory and hippocampal synaptic plasticity. The Erk1/2–CREB signaling pathway may participate in synaptic remodeling (Tan *et al.* 2021*b*) and cognitive function (Kim & Kim 2021) *in vivo* and *in vitro*. The results of the present study showed that testosterone activated the Erk1/2–CREB signaling pathway in the hippocampus of *Tfm* male mice, and Erk1/2 inhibitor SCH772984 blocked the effect of testosterone on upregulating phospho-CREB, PSD95, and GluA1 expression in HT22 cells pretreated with flutamide. It is worth noting that flutamide, as an antagonist of androgen receptors, can largely inhibit the function of androgens, rather than completeness. Consistent with our results, Guo *et al.* (2020) also confirmed that Erk1/2 inhibitor blocked the structural synaptic plasticity of testosterone on primary rat hippocampal neurons. Phospho-CREB may bind to the promoters of susceptibility genes associated with synaptic plasticity to enhance their transcription and then regulate brain functions (Rossetti *et al.* 2022). However, whether Erk1/2–CREB mediates the effect of testosterone on cognitive function and neuroprotection *in vivo* needs to be further demonstrated. In addition to the Erk1/2–CREB signaling pathway, the PI3K–AKT–mTOR signaling pathway in prostate cancer (Cai *et al.* 2020) and cGMP-PKG signaling pathway in the cardiovascular system (Lorigo *et al.* 2020) also may be involved in AR-independent processes; however, their roles on synaptic function need to be further verified.

In addition, the AR-independent action of androgens might be mediated by a membrane-embedded receptor termed the ‘membrane androgen receptor’ (mAR) (Lang *et al.* 2013). Although the structure and function of the mAR have not been fully resolved, four proteins, namely, ZRT-and Irt-like protein 9 (ZIP9), G protein-coupled receptor family C Group 6 member A (GPRC6A), oxoeicosanoid receptor 1 (OXER1), and transient receptor potential melastatin 8 (TRPM8), have been proposed as putative mARs (Thomas 2019, Treviño & Gorelick 2021). It has been reported that testosterone induces apoptosis through ZIP9 in croaker granulosa cells and human breast and prostate cancer cells by activating second messengers (Thomas *et al.* 2018). GPRC6A as a G-protein binding receptor modulated prostate cancer progression is known to be activated by testosterone (Ye *et al.* 2019). OXER1 was involved in the rapid effects of androgens on prostate and breast cancer cells migration by inducing actin polymerization (Kalyvianaki *et al.* 2019). In contrast to other mARs, TRPM8 that had been purified was shown to bind testosterone *in vitro* (Asuthkar *et al.* 2015), and this mAR was involved in the effect of androgens on social behavior (Mohandass *et al.* 2020).

The present study indicates that testosterone enhances the hippocampal synaptic function and spatial memory by activating the Erk1/2–CREB signaling pathway in an AR-independent manner. These findings will provide a new research direction for the effect of androgens within the nervous system.

## Declaration of interest

The authors declare that there is no conflict of interest that could be perceived as prejudicing the impartiality of the study reported.

## Funding

This work was supported by project funding from the National Natural Science Foundation of Chinahttp://dx.doi.org/10.13039/501100001809 (91849134), the Natural Science Foundation of Hebei Provincehttp://dx.doi.org/10.13039/501100003787 (C2020206044, C2020206009), and the Science and Technology Project of Hebei Education Department (QN2020131).

## Author contribution statement

Yizhou Zhang and Meiqin Chen contributed to the experimental phase, methodology, data curation, writing – original draft. Chang Wang and Juan Du contributed to the experimental phase, and Huan Chen, Shixiong Mi, Hongchun Zuo, and Leigang Song contributed to investigation and Visualization. Sha Li and Huixian Cui designed the study; contributed to writing – review, editing; project administration; and funding acquisition.
